# Epithelioid Inflammatory Myofibroblastic Sarcoma Presenting as Gastrointestinal Bleed: Case Report and Literature Review

**DOI:** 10.1097/PG9.0000000000000019

**Published:** 2020-12-17

**Authors:** Alexandra Giannaki, Dimitrios Doganis, Panagiota Giamarelou, Anastasia Konidari

**Affiliations:** From the * B Paediatric Clinic Paidon Aglaia Kyriakou Children’s Hospital; † Paediatric Oncology Department, Paidon Aglaia Kyriakou Children’s Hospital; ‡ Paediatric Pathology Department, Paidon Aglaia Kyriakou Children’s Hospital, Athens, Greece.

**Keywords:** gastrointestinal bleed, gastrointestinal tumor, stomach

## Abstract

Myofibroblastic tumor is a mesenchymal neoplasm composed of myofibroblastic spindle cells with inflammatory infiltrate and considered to be of low-malignant potential tumor. Epithelioid inflammatory myofibroblastic sarcoma (EIMS) is a variant of myofibroblastic tumor with malignant characteristics; it mainly consists of round-to-epithelioid cells with positive nuclear membrane/perinuclear immunostaining for anaplastic lymphoma kinase (ALK) receptor tyrosine kinase. A gastric EIMS case in a 7-year-old boy is discussed. Our patient presented with severe anemia and melena. Magnetic resonance imaging of the abdomen and pelvis revealed a solid tumor (2.7 × 1.9 × 2.6 cm) at the posterior stomach wall. Upper gastrointestinal endoscopy revealed an irregular, protruding, highly vascular, approximately 2 cm mass close to the gastrooesophageal junction. Endoscopic biopsies were taken for histology; tumor cells were epithelioid with eccentric nuclei, prominent nucleoli, and abundant eosinophilic cytoplasm. Immunohistochemistry showed positive staining for desmin, smooth muscle actin, epithelial membrane antigen, cluster of differentiation CD30, and strongly positive staining for ALK. Fluorescence in situ hybridization analysis confirmed the presence of ALK rearrangements. A full-thickness surgical excision of the tumor with clear margins was performed. No adjunct treatment was administered and our patient has remained in full remission at 12 months following the surgery. To the best of our knowledge, this is the first pediatric case of gastric EIMS. Raised awareness and prompt recognition of special histological and immunochemical characteristics of EIMS can lead to accurate diagnosis and targeted therapy.

**What Is Known**

- Epithelioid inflammatory myofibroblastic sarcoma (EIMS) is an aggressive variant of inflammatory myofibroblastic tumor (IMT)
- EIMS usually occurs in children and young adults
- EIMS is mainly located in the abdomen and pelvis

**What Is New**

- Gastric EIMS can present during childhood
- Gastrointestinal bleeding can be the major symptom of gastric EIMS

**I**nflammatory myofibroblastic tumor (IMT) is a mesenchymal neoplasm, which is composed of myofibroblastic spindle cells in a myxoid or collagenous stroma with inflammatory infiltrate, mostly consisted of plasma cells, lymphocytes, and occasionally eosinophils and neutrophils. IMT is common in children and young adults, although a broad age range has been reported. IMT is usually located in the abdomen, pelvis, mediastinum, or retroperitoneum. IMT, which had previously been considered as reactive tumor under the spectrum of “inflammatory pseudotumour,” is now recognized as a distinctive neoplasm of intermediate malignant potential. Recurrence rate ranges from <2% to 25% with metastasis rate of <5% (1, 2).

A rare variant of IMT with specific morphological and immunohistochemical characteristics, was first identified by Marino Enriquez et al and was described as epithelioid inflammatory myofibroblastic sarcoma (EIMS) (3). EIMS is characterized by epithelioid morphology, with nuclear membrane or perinuclear pattern of positive immunostaining for anaplastic lymphoma kinase (ALK); a Ran-binding protein 2 (RANBP2)-ALK fusion is commonly found. ALK activation occurs in all EIMS and 50% of IMTs, a finding which supports the neoplastic origin of the tumor, as ALK protein is known to have an oncogenic role in hematologic and solid tumors (4). EIMS is more aggressive than IMT with recurrence and metastasis rates higher than 80% and 25%, respectively (4, 5).

The recognition of EIMS as a distinct IMT variant is very important because patients with ALK rearrangements can benefit from targeted therapy.

To the best of our knowledge, this is the first case report of gastric EIMS in a 7-year-old boy. Due to its rarity, the characteristics and differential diagnosis are discussed.

## CASE REPORT

**FIGURE 1.**
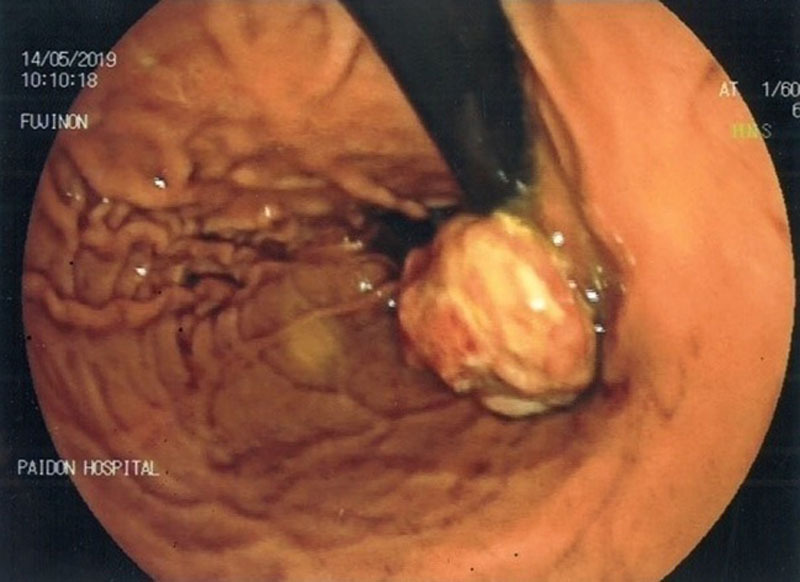
Endoscopic image of the stomach fundus.

A 7-year-old boy presented with melaena and severe anemia (Hb = 4.4 g/dL). Abdominal ultrasound and Meckel’s scan were unremarkable. Upper gastrointestinal endoscopy revealed an irregular protruding highly vascular 2 cm mass close to the gastrooesophageal junction (Figure 1).

**FIGURE 2.**
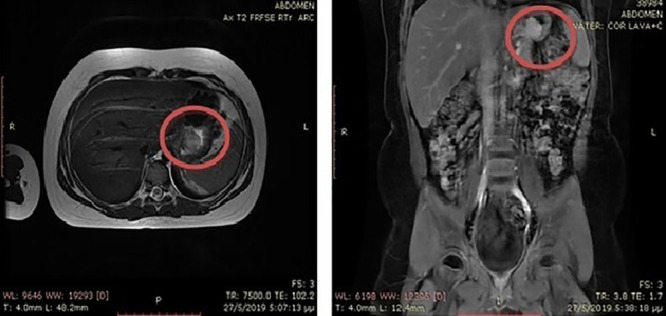
MRI of the thorax and pelvis. MRI = magnetic resonance imaging.

Endoscopic biopsies were sent for histology. Urgent computed tomography of the abdomen and pelvis was performed. Computed tomography revealed a gastric mass and raised the suspicion of lymphoma. Bone marrow examination was within normal limits. Magnetic resonance imaging showed a solid tumor (2.7 × 1.9 × 2.6 cm) at the posterior stomach wall, resembling to a mesenchymal origin tumor, such as a gastrointestinal stromal tumor (GIST) (Figure 2).

**FIGURE 3.**
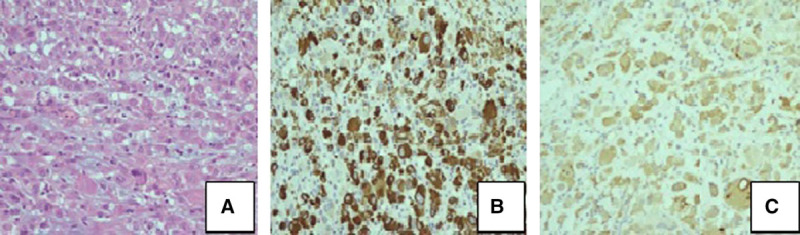
Immunohistochemistry of tumourcells: (a) morphology of epithelioid tumor cells (H/E × 200), (b) positive immunostaining for desmin (Desmin × 200), and (c) ALK positive immunostaining (ALK-1 × 200).

Tumor cells were highly atypical, epithelioid in morphology with pale eosinophilic cytoplasm and irregular nuclei. The cells were distributed in a loose myxoid matrix with a mixed inflammatory infiltrate. Immunohistochemically, the tumor cells were positive for smooth muscle actin, epithelial membrane antigen, desmin and certain cells for CD30 but negative for ALK-1, DOG-1, and for markers of lymphomas or other mesenchymal tumors of childhood (Figure 3). Keeping in mind, the diagnosis of an epithelioid GIST, tumor biopsies were sent to the Harvard Medical School Pathology department for second opinion, where the diagnosis of ALK positive EIMS was made. Fluorescence in situ hybridization analysis confirmed the presence of ALK rearrangements, a finding which highlighted the malignant nature of the tumor and the need for surgical excision.

Following consultation with oncology and surgical departments, a full-thickness surgical excision of the stomach tumor was performed.

The patient did not receive chemotherapy or ALK-targeted therapy because the tumor excision was performed in clear margins, and no evidence of residual disease was found. Follow-up included upper gastrointestinal endoscopy every 3 months thereafter, which has been without pathological macroscopic or histological findings. His blood analysis and magnetic resonance imaging were within normal limits, and the child had no recurrence or metastasis 1 year after surgical excision. A baseline positron emission tomography was also done 3 months after surgery, which revealed no pathology.

## DISCUSSION

A review of the medical literature including Pubmed Central, Web of Science, Scopus, Embase, Google scholar, restricted to publications in English with the medical subject headings IMT, EIMS, tumors, ALK, crizotinib revealed 46 published cases of EIMS reported in the literature until July 30, 2020. Conference proceedings were not included in the analysis. Patient age range varied between 7 months and 76 years (mean age of 32.9 years, male to female ratio close to 2:1) within 11 reported pediatric cases (Table 1). EIMS has mainly been described in the abdomen and pelvis, as shown in Table 1.

**TABLE 1.** Published adult and pediatric EIMS cases by author and anatomic site

Author, reference	Case	Age (A: Adult P: pediatric)	Sex (M: Male F: Female)	Anatomic site
Marino Enriquez et al (3)	1	A	M	Mesentery of the small bowel
	2	A	M	Omentum
	3	P	M	Omentum
	4	A	M	Mesentery of the small bowel
	5	A	M	Mesentery of the small bowel
	6	A	M	Intraabdominal
	7	P	M	Peritoneum
	8	A	M	Peritoneum
	9	A	F	Mesentery of the small bowel
	10	P	M	Omentum and mesentery
	11	A	M	Mesentery of the small bowel
Li et al (6)	12	A	F	Pelvic cavity
	13	A	M	Pelvic cavity
Liu et al (7)	14	A	M	Pelvic cavity
Kimbara (8)	15	A	M	Pelvic cavity
Kurihara Hosokawa (9)	16	A	M	Pelvic cavity
Zhou et al (10)	17	P	M	Intestinal wall and omentum
Wu et al (11)	18	A	F	Pelvic cavity
Bai et al (12)	19	A	M	Colon
Yu et al (13)	20	A	F	Rectum
	21	A	M	Mesentery of ileum
	22	A	M	Mesentery of colon
	23	A	F	Omentum
	24	P	F	Tranverse colon
Jiang et al (14)	25	A	M	Abdominal cavity
Kozu et al (15)	26	A	M	Pleural cavity
Fu et al (5)	27	A	M	Lungs with bone metastases
Du et al (2)	28	A	M	Small intestine and rectum
Xu et al (16)	29	A	M	Abdomen and pelvis
Fang et al (17)	30	P	F	Ovary
Garg et al (18)	31	P	F	Omentum and mesentery
Sarmiento et al (19)	32	A	M	Thoracic cavity
Dermarkarian et al (20)	33	P	F	Orbit
Zhang et al (21)	34	A	F	Abdominal cavity
Telugu et al. (22)	35	P	F	Suprarenal
Xu et al (23)	36	A	F	Stomach
Lee et al (1)	37	A	M	Intraabdominal
	38	A	M	Intraabdominal
	39	A	F	Intraabdominal
	40	P	M	Intraabdominal
	41	A	M	Liver
	42	A	M	Intraabdominal
	43	A	F	Intraabdominal
	44	A	M	Intraabdominal
	45	P	F	Lung
Rafee et al (24)	46	A	F	Multifocal abdominal wall, pelvis, peritoneum

EIMS share the following common characteristics (2): (i) epithelioid tumor cells with round nuclei; (ii) myxoid stroma with inflammatory infiltrates; (iii) ALK positivity with a nuclear membrane or perinuclear staining pattern; (iv) desmin positive immunostaining in the cytoplasm of the tumor cells; and (v) the frequent presence of RANBP2-ALK fusion gene.

In our case report, all those characteristics were identified except the presence of RANBP2-ALK fusion.

ALK is a tyrosine kinase receptor gene located in chromosome 2p23 (2). Rearrangements in the ALK gene result in the expression and overproduction of chimeric fusion proteins. The genetic alterations of ALK are found in various hematologic and stromal malignancies, including lymphomas, lung cancer, neuroblastoma, rhabdomyosarcoma, renal cell carcinoma, inflammatory IMT, inflammatory breast cancer, and melanoma (25). RANBP2-ALK is the most frequent fusion tested to date in cases of EIMS (17). In frame fusion between RANBP2 (2q13) and ALK (2p23) results in continuous ligand-independent ALK autophosphorylation and activation (17). In addition to RANBP2-ALK, RRBP1-ALK fusion, was first reported in 3 cases of EIMS by Lee et al, 2017 (1). ALK fusion is an important clinical biomarker due to the observed clinical improvement of patients with ALK alterations, when treated with ALK inhibitors, such as crizotinib, ceritinib, and alectinib (25).

Diagnosing EIMS can be a challenge due to its unusual round-to epithelioid cell morphology and atypical nuclear characteristics; ALK expression is essential but not pathognomonic. EIMS should be differentially diagnosed from diseases such as follows (2):

i. Anaplastic large cell lymphoma (ALCL): differential diagnosis can be difficult in case of a rare sarcomatoid type of ALCL can exhibit spindle cell morphology with an overlapping immunophenotype, exhibiting positive staining for CD30, ALK, and smooth muscle actin, negative for epithelial membrane antigen and weak desmin expression; RANBP2-ALK fusion protein has not been reported in ALCL to date.
ii. Malignant mesothelioma: MC, CK5, and calretinin stains are positive in immunochemistry analysis, while ALK is absent.
iii. Epithelioid GIST: immunohistochemical stain is positive for CD117, DOG-1, CD34, C-KIT but negative for ALK. In the case of epithelioid GIST, cells have smaller nuclei than those observed in EIMS and there is no inflammation.
iv. Alveolar rhabdomyosarcoma: frequently ALK positive but lacks fibrovascular stroma. Myogenin and MyoD antibodies can lead to its diagnosis.
v. Epithelioid leiomyosarcoma: it often presents with cellular atypia and pleomorphism; however, it lacks the myxoid stroma, inflammatory infiltrate, and ALK expression seen in EIMS.

Surgical resection, where possible, remains the treatment of choice for IMTs/EIMS. Incomplete tumor resection is associated with higher possibility of recurrence (4, 16, 23, 26). Efficacy of second-line treatments such as nonsteroid antiinflammatory drugs, high-dose corticosteroids, biological agents, chemotherapy, and radiotherapy is uncertain (4). ALK inhibitors such as crizotinib may be used in multifocal, refractory disease, and unresectable tumor (4).

The Children’s Oncology Group consortium study which was performed in 79 patients with relapsed or refractory stromal tumors and ALCL estimated the maximum tolerated dose, pharmacokinetics, and toxic effects of crizotinib (between 12 months and 22 years of age, median age 10.1 years) (27). The Children’s Oncology Group study found that crizotinib was well tolerated with a recommended dose of 280 mg/m^2^ twice daily (27). The most common adverse events were neutropenia, liver enzyme elevation, and lymphopenia. This study reported that a targeted ALK inhibitor, such as crizotinib, may be beneficial in childhood malignancies with ALK translocations, mainly in ALCL and IMT (27). If tumor cells are resistant to crizotinib, second generation ALK inhibitors such as ceritinib and brigatinib may be used (28). A second study by the same consortium which was published in 2017 described the outcome of 14 patients (median age 7 years old), with metastatic or inoperable ALK positive IMT who had been treated with crizotinib twice daily. These patients had their disease evaluated using response evaluation criteria in solid tumors (29) and had previously had combined treatment with surgery (n = 8), chemotherapy or antiinflammatory therapy (n = 8). The majority of them showed complete or partial response (12/14) when treated with crizotinib, while in 2/14 the disease remained stable (26).

In conclusion, EIMS is an aggressive variant of IMT, which can present in the stomach and cause upper gastrointestinal bleeding in children. The detection of ALK rearrangements with fluorescence in situ hybridization or RT-PCR is important for establishing the diagnosis. Further research is required in order to explore the benefits and risks associated with ALK-targeted therapy in pediatric EIMS cases.

## ACKNOWLEDGMENTS

The authors would like to acknowledge Dr Maria Kourasi who participated in the technical editing of the manuscript.
